# Skin Cancer Diagnosis Based on Neutrosophic Features with a Deep Neural Network

**DOI:** 10.3390/s22166261

**Published:** 2022-08-20

**Authors:** Sumit Kumar Singh, Vahid Abolghasemi, Mohammad Hossein Anisi

**Affiliations:** School of Computer Science and Electronic Engineering, University of Essex, Colchester CO4 3SQ, UK

**Keywords:** melanoma, neutrosophic, image processing, deep neural network

## Abstract

Recent years evidenced an increase in the total number of skin cancer cases, and it is projected to grow exponentially. This paper proposes a computer-aided diagnosis system for the classification of a malignant lesion, where the acquired image is primarily pre-processed using novel methods. Digital artifacts such as hair follicles and blood vessels are removed, and thereafter, the image is enhanced using a novel method of histogram equalization. Henceforth, the pre-processed image undergoes the segmentation phase, where the suspected lesion is segmented using the Neutrosophic technique. The segmentation method employs a thresholding-based method along with a pentagonal neutrosophic structure to form a segmentation mask of the suspected skin lesion. The paper proposes a deep neural network base on Inception and residual blocks with softmax block after each residual block which makes the layer wider and easier to learn the key features more quickly. The proposed classifier was trained, tested, and validated over PH2, ISIC 2017, ISIC 2018, and ISIC 2019 datasets. The proposed segmentation model yields an accuracy mark of 99.50%, 99.33%, 98.56% and 98.04% for these datasets, respectively. These datasets are augmented to form a total of 103,554 images for training, which make the classifier produce enhanced classification results. Our experimental results confirm that the proposed classifier yields an accuracy score of 99.50%, 99.33%, 98.56%, and 98.04% for PH2, ISIC 2017, 2018, and 2019, respectively, which is better than most of the pre-existing classifiers.

## 1. Introduction

The technical development and scientific innovations in the field of medical science have led to improvement in the surgical condition of a patient; thereby, it has decreased the mortality rate and increased the satisfactory index of patients. Although new discoveries have aided the health care system with advanced diagnosis methods for several diseases, accurate diagnosis and timely treatment of a cancer patient remains exacting and challenging for researchers all around the world. Among several other types of cancers, skin cancer has been the most frequently diagnosed cancer as stated in by the National Institute of Skin Cancer (NISC) [1]. Supporting the statement of NISC, the World Health Organization (WHO) also reported the cases of skin cancer to be exceptionally massive in number, accounting for 1/3rd of the overall cancer cases, which seem to be increasing exponentially with time [2]. Skin being the outermost and massive sense organ of human anatomy is prone to several allergies and fatal infections due to maximum exposure to harmful ultraviolet (UV) radiations from the sun. The skin consists of three successive and overlapped protective layers of the epithelial tissues, namely dermis, epidermis, and hypodermis; these tissues guard the human body against UV radiations. There exists a color pigment termed as melanin, occupying the space at the junction of the dermis and epidermis layers. The melanin is responsible for notable coloration of the iris, skin, and hair. This pigment is produced by cells called melanocytes, based on different factors such as geographical location, climate, and exposure to sun or tanning devices. Melanin is subdivided into two significant categories, namely, eumelanin, which is responsible for the dark color pigmentation, and pheomelanin, which is responsible for the pigmentation of light color. The quantity of each pigment present in the skin varies in every individual, thereby producing various pigmented individuals. People with higher pigmentation levels are less prone to damages and blemishes, which are the adverse effects of prolong exposure to noxious ultraviolet radiations, as stated by the National Institute of Skin Cancer [3]. Therefore, dark pigmented individuals are at low risk of being affected with skin cancer. Overexposure to harsh sunlight might distort the DNA strand, leading to uncontrollable growth of these melanocytic cells. Such deformities can increase the growth of epithelial cells at a rapid rate, thus resulting in tumors or various other skin diseases. At a later stage, the cancerous cell thrives deep into the skin, thereafter damaging vital organs of the human body. Several research studies have revealed that the depletion of the ozone layer is to be blamed for the enormous rise in the number of cases of skin cancer, as it has led to continuous exposure to radiations such a UV-A and UV-B [4]. Skin cancer can be an inherited trait, such as carcinogenic genes present in any of the family members, and it can also occur due to the lack of the pigment called melanin; additionally, it also grows due to sudden exponential growth of dysplastic nevi or benign melanocytic nevi in several parts of the human body.

Skin cancer can be categorized into three major types, i.e., nevus, malignant, and benign [5]. Nevus is merely a birthmark or mole present in the body, which are generally non-melanocytic in nature. Malignant skin cancer is the most deadly and fatal one, as it can spread much faster than the other two types, all over the skin, draining out protein and other nutrition from the neighboring cells, thus rapidly affecting the neighboring cells. The most regularly diagnosed cells of skin cancer patients are Squamous-Cell-Carcinoma (SCC), Malignant Melanoma (MM), and Basal-Cell-Carcinoma (BCC). Both SCC and BCC are non-melanocytic, while MM is inspected to be the most noxious, and it is the reason behind the greatest number of death cases for skin cancer. According to a report of 2019, the statistics conclude that the total number of deaths due to malignant melanoma has touched the mark of 11,650 cases, with 104,350 new cases having been reported in the United States of America [6]. As mentioned by dermatologists, a tumor of malignant lesion, commonly known as melanoma, is an asymmetrical and ameboid-shaped lesion with an irregular border which exists in four to five color shades and of diameter more than 6 mm. Formation of bristles and bleeding from the suspected lesion are a few common traits of malignant lesion. MM usually generates in the dermis layer of the skin and thrives deep into skin and various vital organs very quickly, if not diagnosed and treated on time. Early days of skin cancer diagnosis have marked visual inspection as the only mode of diagnosis, where the suspected lesion was checked by an expert dermatologist via naked eyes by visually comparing normal skin region with tissues of the suspected lesion. This mode of diagnosis has mostly produced false alarms, and thus, a dermosophic imaging tool was developed in the late 1980s, which produces a high-resolution magnified image of the suspected region, thereby enhancing the decision-making procedure. The dermoscopy lens produces the resultant image by filtering reflection over the skin surface [7]. An enhanced and magnified image could be acquired from a dermoscopic imaging tool, which is able to yield an enhanced score of accuracy for diagnosis of skin lesion as compared to purely visual inspection; however, the dermatologist still faces problems in decision making due to visual similarity of melanoma and benign cancers. Therefore, it could be concluded that manual inspection of the skin lesion is subjective, time-consuming, error-prone, and complex, and thus, a fully automatic and reliable mode of diagnosis i.e., computer aided diagnosis (CAD), is introduced in recent decades, which not only produces a higher score for sensitivity and specificity, but also aid the dermatologist while classifying suspected lesion. CAD systems are widely accepted by researchers around the globe for classification of melanoma lesion. Several deep learning models are proposed for the accurate classification of skin lesions [8]. Ref. [9] proposed an automatic system for classification of melanoma using deep convolutional neural networks. The acquired image is digitally pre-processed before undergoing the classification phase, which increases accuracy of classification. Ref. [10] employed the method of generalized class of fractional partial differential equations for enhancement of skin lesion. A pixel’s fractional mean-based image enhancement algorithm was employed by [11] for better image splicing detection, which enhances the quality of image, thereby enabling the classifiers to detect more features. Ref. [12] proposed a method for diagnosis of skin cancer using convolutional neural network for smartphone, which marks a significant growth in the field of digital diagnosis of melanoma. Ref. [13] used ESRGAN for pre-processing of skin lesion; thereafter, they experimented with several CNN models such as Resnet 50, Inception net, and Inception Resnet. 

In this article, we have proposed a novel computer-aided diagnosis system (CAD) for digitally diagnosing the suspected lesion. A novel and effective pre-processing phase is introduced in the article which not only removes the artifacts (such as hair follicles, blood vessels, dermoscopic ruler, and frames) and reflections from the acquired images, but also enhances the image and automatically adjusting contrast and brightness of the image. Pre-processing is the fundamental and most important phase of CADs as availability of artifacts, such as hair follicles and blood vessel, affect the visual inspection of the lesion and hamper the accuracy of diagnosis, and thus, digitally removing such artifacts not only reduces the pain and labor of removing those artifacts over suspected lesion but also enhances the diagnosis outcome. Inspired from uncertainty theory, we want to incorporate the vagueness principle into image segmentation techniques to discard the ambiguity portion of a segmented image and to capture the best fitted region more efficiently. Obviously, some crucial questions to be asked are, if the size of the image is large, how we can cut or capture the desired actual feasible region in a logical way using the pixel values? Additionally, in case of segmentation how we can capture the hesitation portion using uncertainty logic? Most importantly, how can we link the matrix presentation of a segmented image with the pixel values? In case of iteration process where mathematical theory will be utilized to get the original affected region? This research article deploys a determinant based image segmentation method namely absolute value computational algorithm using each pixel values for three channels to detect the most affected region from the captured image. Initially, we will follow an algorithm based on 3 × 3 determinant constructions of the segmented image using the pixel values. We will scale down the total image into finite number of determinants and will calculate all the absolute values for each of the three channels. Furthermore, we will set the threshold value using the geometric mean concept, which will capture the affected region roughly from the original figure. After that, we will incorporate neutrosophic theory to judge the exact affected portion after 1st phase of segmentation. Neutrosophic number can grab all three components of an uncertain number, namely truth, false, and hesitation portions, very efficiently and logically. Here, we can utilize the pentagonal linguistic neutrosophic number for the second phase of segmentation to set the exact threshold value such that the image segmentation process can be performed properly to get more accurate approximation. Then, the article utilizes the segmented region for classification of lesion using proposed classifier in the Keras [14] framework. The model is trained over the PH2 [15], ISIC 2017 [16], ISIC 2018 [17], and ISIC 2019 [18] datasets of dermoscopic images. A huge set of dermoscopic data along with dense layers of classifiers yields an effective score for sensitivity and specificity. The proposed segmentation and classification method is evaluated for four publicly available datasets: PH2, ISIC 2017, ISIC 2018, and ISIC 2019. The first two have three categories of skin lesion images including melanoma, while the last two datasets have seven and eight categories, respectively. Moreover, the increase in classification accuracy have been marked due to various data tuning technique, such as data augmentation and balancing. The experimental results also depict the importance of segmentation phase prior to classification, as it plays a vital role in enhancement of the accuracy score. The diagnostics performance is critically being affected by the methods of data augmentation, rebalancing, and pre-segmentation. This article focuses on proper rebalancing the training data such that performance of diagnosis could be enhanced. The pre-segmentation phase is also considered to be one of the prerequisite steps, as the important features is targeted during classification of image and the irrelevant features of the surrounding tissues/skin (field of view) are segmented out of the lesion images. We have proposed a neural network, which employs inception and residual blocks with a SoftMax layer after each residual black, which makes the network wider; this paper addresses the classification performance by various well known deep learning classifiers and states that the proposed network achieves highest score for accuracy. The results achieved by this article provides a guideline for application of CAD system.

The fundamental motive of this research article is to develop a trustworthy CAD method based on principles of neutrosophy and deep learning for precise segmentation and classification of skin lesion. The forthcoming section in this article portrays materials and methods in Section 2, where the preliminaries to neutrosophic number is highlighted along with pre-processing using novel methods for removal of digital artifacts and image enhancement, segmentation using neutrosophy, and proposing a novel architecture for classification of the lesion. The experimental result is presented in Section 3. The paper ends with a discussion and conclusion in Section 4 and Section 5, respectively.

## 2. Materials and Methods

**Figure 1:**
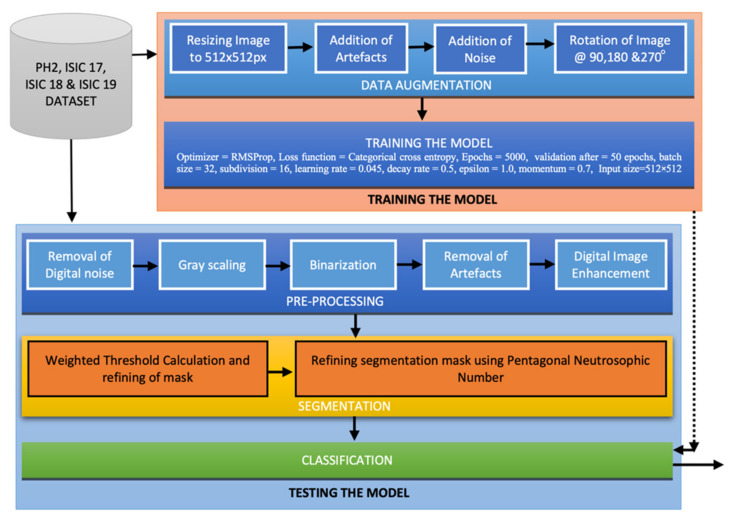
Pictorial representation of the proposed method.

This section explains the proposed method in detail, which uses different stages of computer aided diagnosis, namely: pre-processing, segmentation, lesion localization, and classification. Novel methods for pre-processing of lesion is introduced in this paper, which not only enhances the image quality but also digitally removes the artifacts. A unique segmentation phase is proposed in the article using neutrosophy and determinant to calculate the threshold value for segmentation of the lesion. Thereafter, a modified classifier is employed for classification of skin lesion into a malignant and non-malignant class. A complete flowchart of the proposed method is pictorially represented in Figure 1. The training dataset from PH2, ISIC 2017, ISIC 2018, and ISIC 2019 repositories are used to train the proposed deep neural network. Training data are augmented before passing it to the neural network. Training data being of different resolution is difficult to train on a neural network; thus, the images are resized into 512 × 512 pm. Thereafter, digital artifacts such as hair follicles, dermoscopic ruler and frame mark and mark of blood vessels are digitally added to the training image such that the accuracy of diagnosis in the real world (for holdout datasets) increases. Digital noise is also added to each of the images such that the model performs well in a real-world scenario. Henceforth, the images are rotated at an angle of 90, 180, and 270 degrees; therefore, the training dataset is augmented to four times is original size. Then, the augmented images are passed into the proposed neural network with specific hyperparameters (more about the proposed model and hyperparameters is mentioned in Section 2.4.1 Implementation and training). The test data from the public repositories of PH2, ISIC 2017, ISIC 2018, and ISIC 2019 are used to assess the performance of the model. The test data are pre-processed by the proposed pre-processing method, which includes the removal of digital noise and artifacts. In the next stage, the image is enhanced by the process of histogram equalization. The pre-processed image undergoes the segmentation phase, where the lesion is accurately segmented using the algorithms of weighted threshold calculation, which is followed by neutrosopic-based threshold calculation of the image but segmentation of lesion. Finally, the dermoscopic lesion image is classified to be either a melanoma or non-melanoma using the proposed model.

### 2.1. Mathematical Preliminaries

**Neutrosophic Set [19]:** A set $\tilde{A_{Nue}}$ is termed as a neutrosophic set if $\tilde{A_{Nue}}=\{(x;[\omega_{\tilde{A_{Nue}}}\left(x\right),\;\sigma_{\tilde{A_{Nue}}}\left(x\right),\;\eta_{\tilde{A_{Nue}}}\left(x\right)])\;\vdots \;x\in \;X\}$, where $\omega_{\tilde{A_{Nue}}}\left(x\right):X\to ]0^{-},1^{+}[$ is termed as the truth function, $\sigma_{\tilde{A_{Nue}}}\left(x\right):X\to \left[0^{-},1^{+}\right]$ is called the hesitant function, and $\eta_{\tilde{A_{Nue}}}\left(x\right):X\to \left[0^{-},1^{+}\right]$ is called the falsity function.

Additionally, $\omega_{\tilde{A_{Nue}}}\left(x\right),\;\sigma_{\tilde{A_{Nue}}}\left(x\right)\;\&\;\eta_{\tilde{A_{Nue}}}\left(x\right)$. satisfy the following the relation:

(1) $$0^{-}\leq Sup\{\omega_{\tilde{A_{Nue}}}\left(x\right)\}+Sup\{\;\sigma_{\tilde{A_{Nue}}}\left(x\right)\}+Sup\{\;\eta_{\tilde{A_{Nue}}}\left(x\right)\}\leq \;3^{+}$$

**Single-Valued Pentagonal Neutrosophic Number (SVPNN) [20]:** A SVPNN $\left(\tilde{S}\right)$ is defined as $\tilde{s}=\langle \left[\left(a^{1},b^{1},c^{1},d^{1},e^{1}\right);\pi \right],\left[\left(a^{2},b^{2},c^{2},d^{2},e^{2}\right);\rho \right],[(a^{3},b^{3},c^{3},d^{3},e^{3});\sigma ]\rangle$, where $\pi ,\rho ,\sigma \in \left[0,1\right]$. The truth function $\left(\tau_{\tilde{S}}\right):\mathbb{R}\to \left[0,\pi \right]$, the hesitation function $\left(\vartheta_{\tilde{S}}\right):\mathbb{R}\to \left[\rho ,1\right]$, and the falsity function $\left(\varepsilon_{\tilde{S}}\right):\mathbb{R}\to \left[\sigma ,1\right]$ are given as:

(2) $$\tau_{\tilde{S}}\left(x\right)=\left\{\begin{array}{cc} \frac{\pi \left(x-a^{1}\right)}{\left(b^{1}-a^{1}\right)} & a^{1}\leq x<b^{1}\\ \frac{\pi \left(x-b^{1}\right)}{\left(c^{1}-b^{1}\right)} & b^{1}\leq x<c^{1}\\ \pi & \begin{array}{c} x=c^{1} \end{array}\\ \frac{\pi \left(d^{1}-x\right)}{\left(d^{1}-c^{1}\right)} & c^{1}\leq x<d^{1}\\ \frac{\pi \left(d^{1}-x\right)}{\left(e^{1}-d^{1}\right)} & \;d^{1}\leq x<e^{1}\\ 0 & otherwise \end{array}\right.\;\;$$

(3) $$\vartheta_{\tilde{S}}\left(x\right)=\left\{\begin{array}{cc} \frac{b^{2}-x+\mu \left(x-a^{2}\right)}{\left(b^{2}-a^{2}\right)} & \;a^{2}\leq x<b^{2}\\ \frac{c^{2}-x+\mu \left(x-b^{2}\right)}{\left(c^{2}-b^{2}\right)} & b^{2}\leq x<c^{2}\\ \mu & \begin{array}{c} x=c^{2} \end{array}\\ \frac{x-c^{2}+\mu \left(d^{2}-x\right)}{\left(d^{2}-c^{2}\right)} & c^{2}\leq x<d^{2}\\ \frac{x-d^{2}+\mu \left(e^{2}-x\right)}{\left(e^{2}-d^{2}\right)} & \;d^{2}\leq x<e^{2}\\ 1 & otherwise \end{array}\right.\;\;$$

(4) $$\varepsilon_{\tilde{S}}\left(x\right)=\left\{\begin{array}{cc} \frac{b^{3}-x+\sigma \left(x-a^{3}\right)}{\left(b^{3}-a^{3}\right)} & \;a^{3}\leq x<b^{3}\\ \frac{c^{3}-x+\sigma \left(x-b^{3}\right)}{\left(c^{3}-b^{3}\right)} & b^{3}\leq x<c^{3}\\ \sigma & \begin{array}{c} x=c^{3} \end{array}\\ \frac{x-c^{3}+\sigma \left(d^{3}-x\right)}{\left(d^{3}-c^{3}\right)} & c^{3}\leq x<d^{3}\\ \frac{x-d^{3}+\sigma \left(e^{3}-x\right)}{\left(e^{3}-d^{3}\right)} & \;d^{3}\leq x<e^{3}\\ 1 & otherwise \end{array}\;\right.\;$$

where $-0\leq \tau_{\tilde{S}}\left(x\right)+\vartheta_{\tilde{S}}\left(x\right)+\varepsilon_{\tilde{S}}\left(x\right)\leq 3+,x\in \tilde{S}$.

### 2.2. Pre-Processing

In order to remove noise from the acquired input image *f*[*x, y*], we have applied a filter $n_{\sigma_{s}}$ on the image *f*[*x,y*], such that noise at pixel (*i, j*) is flattened.:

$$Filter=n_{\sigma_{s}}\left[i-x,j-y\right]$$

where:

(5) $$n_{\sigma_{s}}\left[x,y\right]=\frac{1}{2\pi \sigma_{s}^{2}}e^{-\frac{1}{2}\left(\frac{x^{2}+y^{2}}{\sigma_{s}^{2}}\right)}$$

Thus, we get a modified image *m*[*i,j*] by applying the filter over the image, which is:

(6) $$m\left[i,j\right]=\frac{1}{W_{b}}\sum_{x}\sum_{y}f\left[x,y\right]n_{\sigma_{s}}\left[i-x,\;j-y\right]$$

In order to maintain the energy in the filter = 1, $W_{b}$ (weighting function) is created in order to add the product of spatial filter and brightness filter:

(7) $$W_{b}=\underset{x}{\sum }\underset{y}{\sum }n_{\sigma_{s}}\left[i-x,\;j-y\right]n_{\sigma_{s}}\left(f\left[x,y\right]-f\left[i,j\right]\right)$$

However, due to the variation in the intensity of the image, a global filter alone cannot be employed to reduce digital image noise. Moreover, the image is smoothened to a great extent with an increased value of $\sigma_{s}$, which might lose a few important features of the image and a low value of $\sigma_{s}$ might not be effective in the process of noise removal. Thus, a dynamic filter is required for enhanced noise filtering. The proposed filter works on the intensity value of the image and the filter is modified for each pixel. If the modular difference between the center [*m,n*] and [*I, j*] is more than $L+s\frac{\left(f_{m}-f_{p}\right)}{\left(f_{m}-f_{p}\right)+\left(f_{m}-f_{s}\right)}-\frac{\sum f_{i}}{s}$, then [*i,j*] is modified to 0 to avoid obsoletion of features. The modified brightness filter can be shown as:

(8) $$n_{\sigma_{b}}\left[a\right]=\frac{1}{\sqrt{2\pi \sigma_{b}}}e^{-\frac{1}{2}\left(\frac{k^{2}}{\sigma_{b}^{2}}\right)}$$

Thus, our modified equation for removal of extra unwanted noise is:

(9) $$m\left[i,j\right]=\frac{1}{W_{b}}\sum_{x}\sum_{y}f\left[x,y\right]n_{\sigma_{s}}\left[i-x,\;j-y\right]n_{\sigma_{b}}\left(f\left[x,y\right]-f\left[i,j\right]\right)$$

As mentioned in Equation (9), digital noise is removed, and the image is smoothened without losing the key features which might be lost if we had only employed a Gaussian filter.

Artifacts such as presence of hair follicles (both thin and thick), presence of blood vessels, and reflection of dermoscopic gel and dermoscopic frames are digitally removed by the proposed method, where the noise free image $m{\left[i,\;j\right]}_{3}$ is converted into $m{\left[i,\;j\right]}_{1}$, which is a monochromatic image. Henceforth, *m*[*i,j*] is binarized and it was checked if the selected pixels are continuous or discrete. Continuous and regular block of mask are not considered to be hairs follicles and they are set as the background of the mask. Thereafter, the binarized mask is alternatively diluted and convolved to form prominent lines of hair, which could be masked out from the original image *f*[*x,y*]. This method of hair removal also enables to remove marks from the dermoscopic ruler and frames. 

**Figure 2:**
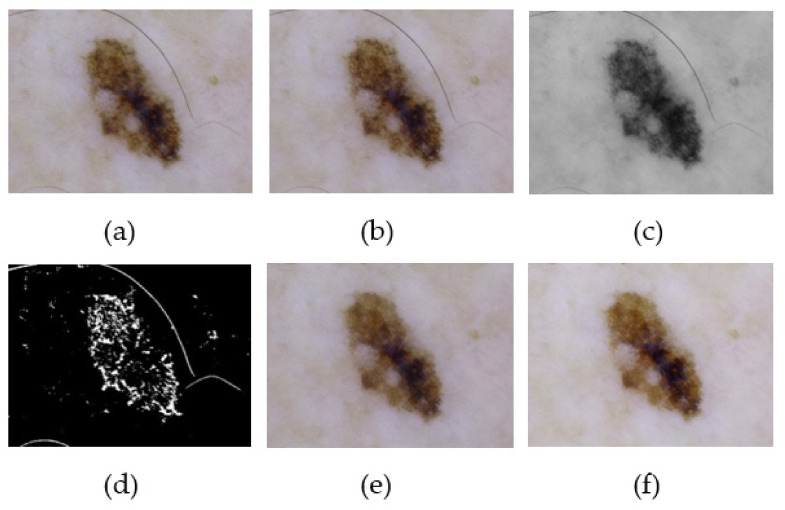
Illustration of the proposed pre-processing phase. (**a**) Acquired image. (**b**) Removal of digital noise. (**c**) Gray scaling of image. (**d**) Binarization of image. (**e**) Removal of artifacts. (**f**) Digitally enhanced image.

Finally, the pre-processed image undergoes the process of image enhancement by histogram equalization to adjust the brightness and contrast of each pixel. The enhanced image is further used for lesion localization and segmentation. Additionally, the efficiency of the proposed method is illustrated in the result and analysis section, which clearly depicts the efficiency of the proposed pre-processing method. Figure 2 represents the pre-processing of the dermoscopic lesion at different proposed stages.

### 2.3. Segmentation

Initially, the pre-processed image $\left(I\right)$ of dimension $\left(x,\;y\right)$ is unified into a single channel, where:

(10) $$I_{i,j}^{new}=\sqrt[{3}]{I_{i,j}^{r}\times I_{i,j}^{g}\times I_{i,j}^{b}}$$

Thereafter, $I_{min}\;and\;I_{max}$ are calculated from $I_{i,j}^{new}$, which is encapsulated along with weight w1 and w2 to calculate global threshold (γ) of the image:

(11) $$\gamma =\frac{\left(I_{min}\times w1\right)+\left(I_{max}\times w2\right)}{\left(w1+w2\right)}$$

where w1 and w2 are 0.4 and 0.6, respectively. To form a segmentation mask, if $I_{i,j}^{new}<\gamma$, the pixel is part of mask $\left(M_{\left(i,j\right)}=255\right)$, otherwise it is discarded $\left(M_{\left(i,j\right)}=0\right)$. Furthermore, the mask $\left(M\right)$ is convolved with a filter $\left(F_{1}\right)$ of size 3×3, which is iterated over M to calculate the minima of overlaying pixels, thereafter changing the $F_{1_{\left(1,1\right)}}$ to minimal value. For, each stride of filter, the new mask can be algorithmically represented as: $F_{1_{\left(1,1\right)}}=\left(\sum M_{\left(i+3,j+3\right)}-\sum F_{1}\right)?255:0$. Thereafter, the filtered mask is convolved with filter $\left(F_{1}\right)$ of size 7×7, it is iterated over a filtered mask, and maxima of overlaying pixels are calculated and assigned to $F_{1_{\left(1,1\right)}}$, where $F_{1_{\left(1,1\right)}}=M_{\left(i+3,j+3\right)}?255:0$. 

**Figure 3:**
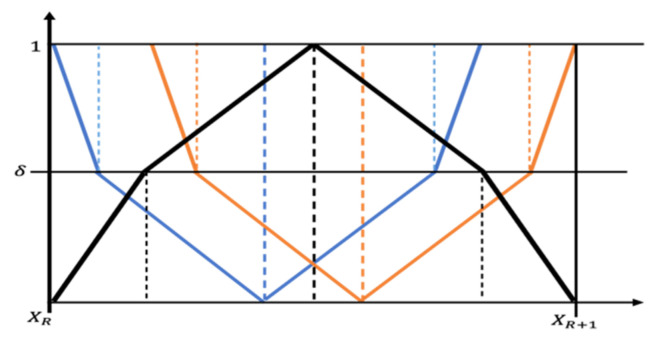
Pictorial representation of a pentagonal for different neutrosophic numbers.

Henceforth, the segmentation mask is formed, but we can observe a fuzziness in boundaries of the segmented lesion. We are confused in case of the next phase of segmentation, as all the pixel values lie within a small bandwidth. Moreover, we are in a dilemma which pixel should be captured and which one should be discarded. Thus, for the next phase of segmentation, we will utilize the linguistic pentagonal neutrosophic number to select the threshold value of the next phase. Structure of PNN is pictorially represented in Figure 3, where false, hesitance, and truth value are represented on the *y*-axis as 0, δ, and 1, respectively. $X_{n}\;and\;X_{n+1}$ represents the possible range of threshold for segmentation of skin lesion. The neutrosophic threshold is an accurately determined probabilistic point between this range. We know that any neutrosophic number can grab degree of true, false, and indeterminacy value of a membership function in a compact way. 

***Case-1:*** If $\left|X_{i}\right|<\vartheta$, where $\left|X_{i}\right|$ is the pixel value of the *i*th pixel of the segmented image, then the pixel is discarded.

***Case-2:*** If $\left|X_{i}\right|\geq \vartheta$, where $\left|X_{i}\right|$ is the pixel value of the *i*th pixel of the segmented image, then the pixel is accepted for the next round.

**Figure 4:**
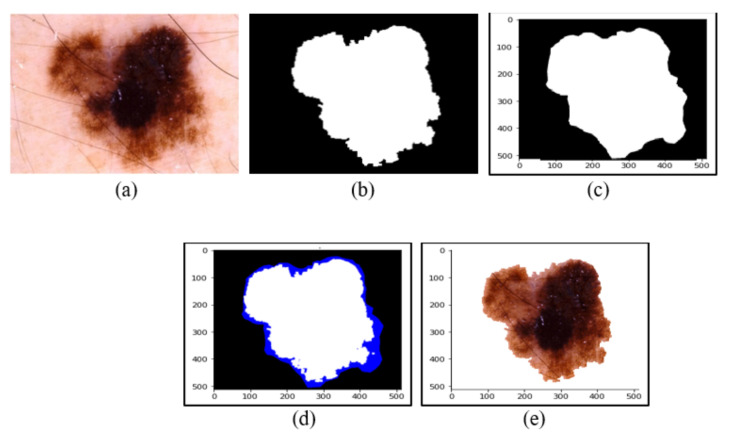
Segmentation of the dermoscopic image by the proposed method. (**a**) Acquired image. (**b**) Ground truth segmentation mask. (**c**) Segmented mask of skin lesion after the first phase. (**d**) Segmented mask of skin lesion after second phase. (**e**) Final segmented portion of skin lesion.

The conception of neutrosophic number is being proposed here to tackle the ambiguity portion and to fix the threshold value ν. A linguistic pentagonal neutrosophic number is capable to define all the three components of an uncertain number of (i) true, (ii) false, or (iii) hesitation, so in this circumstance, a pentagonal neutrosophic number (PNN) is proposed to tackle the threshold value computation. In case of hesitation, the PNN successfully generates a threshold value, which supports the segmentation method to yield a high score of segmentation. Therefore, the same concept is applied to set the threshold value T. Thus:

(12) $$\vartheta =\frac{1}{15}\left(m_{1}+m_{2}+m_{3}+m_{4}+m_{5}\right)\times \left(2+\pi -\sigma -\mu \right)$$

where $\pi ,\sigma ,\mu \in \left[0,1\right]$, ($m_{1},m_{2},m_{3},m_{4},m_{5}$) represents the pentagonal neutrosophic components and π indicates the truth, σ indicates the indeterminacy, and μ indicates the falsity part of the membership function. The asymmetrical PNN is also considered, as in a real-time situation, the threshold value may not always be symmetrical PNN. Here, we utilized the linguistic PNN such that it can grab all the verbal information (Very low, Low, Median, High, Very High) in a compact way and no other structure can grab this idea. Figure 4 represents the segmentation performance of proposed method, where a dermoscopic image and its respective ground truth segmented mask is compared with the proposed segmentation masks to portray the accuracy of the proposed model. It can be easily concluded from Figure 4 that the first segmentation phase draws a rough outline across the lesion, which is refined in the second phase using the pentagonal neutrosophic number.

### 2.4. Classification

Classification is the fundamental phase of computer-aided diagnosis, where the acquired skin lesion image is classified to be malignant or non-malicious by using our proposed and efficient deep learning model. It is trained over a publicly available and standard dataset of ISIC. The forthcoming subsections deal with a detailed description of datasets that are employed for training and the hyperparameter used to fetch the best-fit results of classification.

#### 2.4.1. Implementation and Training

With the advancement in medical vision and computer-aided diagnostic systems, the classification of a malignant lesion has significantly improved. However, training a classifier to detect a particular class is a strenuous task. The ISIC 2017, ISIC 2018, and ISIC 2019 datasets are used for training the classifier, with a total of 103,524 images, out of which 15,464 are melanoma images, while 88,060 belong to non-malignant images. A huge set of training data increased the efficiency of the classification. To unify the dataset to overcome the problem of classification for various images of multiple dimensions, the training images are resized to 512×512 pixels. Additionally, this resizing of the dataset also decreases the computational processing of skin lesion classification, thereby increasing the training speed. The training data along with its ground truth result are passed through the proposed model of a deep neural network. 

**Figure 5:**
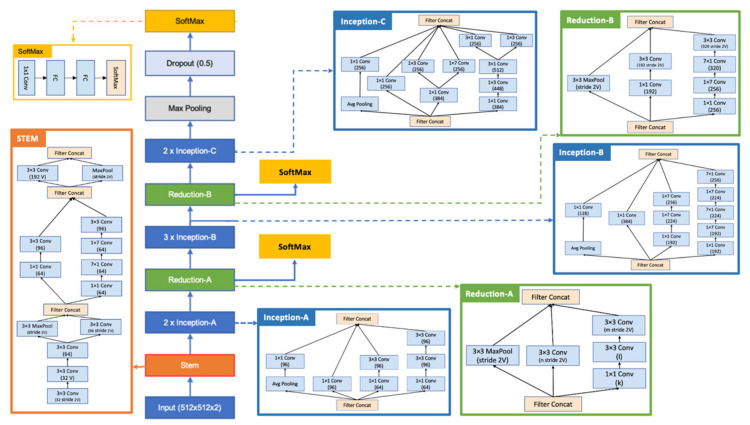
Flowchart of the proposed neural network.

This article proposes a classifier based on the inception and residual block with a softmax block after each residual block, which makes the network wider and less deep, thereby decreasing the training time. Pictorial representation of the proposed neural network is shown in Figure 5. The network takes an input image of shape 512 × 512 px and thereafter passes it through Stem block, which is inspired from the Inception Resnet V2. It consists of series of parallel convolution layers which are concatenated to preserve the key features of the image. The Inception blocks (A, B and C) contains a series of 1 × 1 and 3 × 3 convolutional layers along with average pooling layer, such that both minor and major features are put to attention. The dimension of the image is kept like its input size, and at the end of each inception block, the filters are concatenated to produce more kernels of the same dimension. The reduction blocks (both A and B) are used to reduce the dimension of the image without losing any of the key features. The softmax block comprises of a 1 × 1 convolutional layer and two fully connected (FC) layers, followed by a softmax layer for the classification of lesion. This block is attached after every reduction unit, thereby helping in the optimization of weight and biases on the network at each stage. This technique of updating weight and biases prior to final classification helps the network to learn faster and enables it to produce a high score for accuracy. A max pooling layer is used after the Inception-C block to reduce the size of the network and keep the key value of the image; thereafter, a dropout of 50% is applied to reduce the filter size, as most of the filters might be repetitive as we have used higher value of small filters. Images from ISIC public directory are grouped into two different classes, i.e., melanoma and non-melanoma, and they are passed as the only parameter to the neural network and based on the convolution images are classified to be of either of the class. The proposed architecture of the neural network seems to produce a high value of accuracy for the classification of a melanoma lesion.

The proposed classifier fetches the best fit results for classification under the following set of hyperparameters: batch size = 32, subdivision = 16, learning rate = 0.045, decay rate = 0.5, epsilon = 1.0, and momentum = 0.7. The classification result is generated for 5000 epochs while saving results for each 50th epoch. This criterion is evaluated to fetch enhanced classification results, as the learning rate is increased by saving epochs after 50th phase. The RMSProp optimizer is used for updating weight and biases during backpropagation and categorical_crossentropy is used as a loss function, as it seems to perform better with ‘RMSProp’. The classifier is validated after each 50th epoch to check the enhanced efficiency of lesion detection. The configuration file is generated after training over a huge dataset of ISIC and is used to classify skin lesion images of the test dataset. The optimal hyperparameters are selected by employing Bayesian optimization methods. The key benefit of Bayesian approaches is that they can inform the choice of the subsequent hyperparameter values to be evaluated by using the results from historical runs or gradually increasing prior knowledge in the form of pairs of hyperparameter values and objective function scores. This reduces the time and computation costs required to attain acceptable objective function scores.

### 2.5. Augmentation of the Dataset

**Figure 6:**
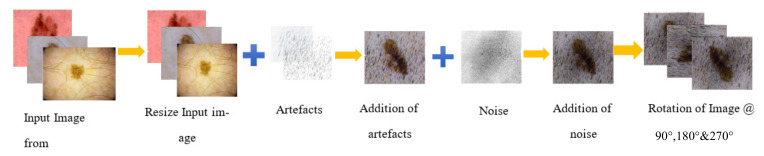
Representation of data augmentation techniques employed in this article.

Efficient processing of dermoscopic images and accurate classification of skin lesions has emerged as a vital field of research. The accumulation of relevant datasets and accurate training of the classifier under specific parameter has always being a perplexing job. The proposed method is trained over the publicly accessible datasets of ISIC 2017, ISIC 2018, and ISIC 2019. These datasets consist reliable and easily available ground truth images that were drawn by a panel of expert dermatologist, which enable the researchers to compare and evaluate the proposed methodology. A huge set of dermoscopic data (after the process of data augmentation) not only helps the classifiers to train under rigorous conditions, but also increases the validation size, thereby producing an enhanced report of classification. The augmentation of data produces more training images from the same sample set by addition of digital noise and artifacts and rotating the image at specific angles, therefore helping the model to learn more features and classify the skin lesion accurately. Refs. [21,22] employed the process of data augmentation to increase the sample size of skin lesion image, thereby increasing the performance of the proposed model. Table 1 gives a summarized figure of total number of dermoscopic images for each dataset into training, testing, and validation, respectively. The pedro hispano hospital (PH2) dataset is a well-known dermoscopic dataset which is obtained from the Hospital of Pedro hispano, and it contains 200 dermoscopic 8-bit RGB images that are 761 × 570 to 769 × 577 pixel in dimension. It consists of 80 common nevi, 80 atypical nevi, and 40 melanoma images; this dataset is used as a hold-out dataset (used only for testing the performance of segmentation and classification) in our article. The ISIC dataset of 2017 was developed by the International Symposium on biomedical images (ISBI) organization. It contains three classes: Benign Nevus (BN), Seborrhoeic Keratosis (SK), and Malignant melanoma (MM). Image resolution of both training and testing images is ranging from 1022 × 767 to 6748 × 4499 pixels; these are 8-bit RGB images. The ISIC 2018 dataset has sources from HAM10000, and it contains 10,015 training images along with ground truth values and metadata, which contains the classification results. We have segregated 10,015 training images into 8695 images for training and 1320 dermoscopic images for testing the classifier. The ISIC 2018 dataset contains seven classes of different skin diseases. The ISIC 2019 dataset constitutes of 25,311 8-bit RGB images in the training dataset, of which 60% is used for training and 20% each for testing and validation of the proposed method. The dataset contains metadata and ground truth images for training; thus, only the training dataset is used in this research for training the classifier and evaluating it, such that we will get a result to compare our values. The dataset is derived of eight different classes: Squamous cell carcinoma, Vascular Lesion, Dermatofibroma, Benign Keratosis (Lichen planus-like keratosis, seborrheic keratosis and solar lentigo), Actinic Keratosis, Basal Cell Carcinoma, Melanocytic Nevus, and Melanoma. Each image of the dataset is resized to 512 × 512 pixels to reduce the dimensionality, such that we can perform the image enhancement and classification easily at less expensive computational cost. Henceforth, extra artifacts, such as thick and thin hair, ruler marks, and unwanted reflections and noise, are added to these replicated images, such that it can train the classifier to fetch enhanced results under any condition. Augmentation of the dataset at various angles is performed to produce a modified classification outcome, even for images acquired at a different angle. The sum of each training dataset is four times the original count, as each dermoscopic image is rotated at an angle of 90°, 180°, and 270°. The total count of dermoscopic images for training the classifier was 25,881, which is increased to 103,524 after replication of the training dataset. Therefore, a total of 115,899 dermoscopic images are used for training, testing and validation in this research article. Pictorial representation to data augmentation is illustrated in Figure 6.

### 2.6. Performance Evaluation Metrics

This section specifies different evaluation metrics that are used to validate and evaluate each stage of the CAD system: pre-processing, localization, segmentation, and classification. These metrics are accepted and employed globally by well-known researchers in similar domains.

#### 2.6.1. Evaluation Metric for Pre-Processing of Lesion

The efficiency of the pre-processing stage is evaluated by employing the universal image quality index (UIQI), peak signal to noise ratio (PSNR), root mean squared error (RMSE), and mean squared error (MSE). These matrices are used to calculate the enhancement capacity of the dermoscopic image in pre-processing stage, which might increase the efficiency of lesion detection in later stages. These parameters also measure the efficiency of the pre-processing method by measuring its capacity to remove artifacts digitally. The mean difference between an acquired image with noise *I*(*x,y*) and a pre-processed image *Is*(*x,y*) is calculated by the MSE. The square root of the MSE is the RMSE. The equations for both the MSE and RMSE are given as follows:

(13) $$MSE=\frac{1}{mn}\overset{m-1}{\underset{i=0}{\sum }}\overset{n-1}{\underset{j=0}{\sum }}{\left[I_{s}\left(i,j\right)\;–\;I\left(i,j\right)\right]}^{2}$$

(14) $$RMSE=\sqrt{\frac{1}{mn}\overset{m-1}{\underset{i=0}{\sum }}\overset{n-1}{\underset{j=0}{\sum }}{\left[I_{s}\left(i,j\right)\;–\;I\left(i,j\right)\right]}^{2}}$$

The quality of image is widely measured by the peak signal to noise ratio (PSNR). The quality of image enhancement is marked by a high PSNR score. It is usually expressed in terms of the logarithmic decibel (dB) scale. The mathematical representation of the PSNR is given below in Equation (15):

(15) $$PSNR=-20\;x\;log_{10}(\frac{I_{s\left(max\right)}}{MSE})$$

*UIQI* is the estimation of linear correlation of acquired dermoscopic image along with the pre-processed image, based on luminance, contrast, and structure features in the pre-processing stage. $\bar{I}$, $\bar{I_{P}}$, and *σ* represents the mean of the input image, mean of pre-processed image, and standard deviation of the acquired image. The mathematical representation *UIQI* is illustrated below:

(16) $$UIQI=\frac{4\sigma_{I_{P},I}\bar{I_{P}}\bar{I}}{\sigma_{I_{P}}^{2}+\sigma_{I}^{2}\;\left[\bar{I_{P}}{}^{2}+{\bar{I}}^{2}\right]}$$

#### 2.6.2. Evaluation Metric for Localization of Lesion

The performance of an algorithm is visualized by a 2 × 2 table, which is known as a confusion matrix or error matrix. It encapsulates a detailed report of prediction results on a classification problem. Each row represents an occurrence of the predicted class and each column indicates occurrence of actual class. It is employed for localization and classification of malignant lesion. If a malignant lesion is predicted to be malignant, it is defined as a true positive (*TP*) value, whereas if it is predicted to be non-malignant, it is defined as a false negative (*FN*). If a non-malignant lesion is predicted to be melanoma, it is classified as a false positive (*FP*), as it raises a false malignant flag, whereas if it is predicted as non-malignant, the classification is defined as a true negative (*TN*).

Evaluation of melanoma lesion localization is executed by an overlapping predicted region over the ground truth region to find the intersection area; this greedy method is termed as intersection-over-union (*IoU*), which is represented by Equation (17):

(17) $$IoU=2*\frac{\;TP}{TP+FN+FP}=\frac{Area\;of\;overlap}{Area\;of\;union}$$

Mean average precision (mAP) is used for evaluating localization phase by computing the mean precision of detection of melanoma area. Equation (18) shows the detailed mathematical representation:

(18) $$mAP=mean\frac{TP}{TP+FP}$$

#### 2.6.3. Evaluation Metric for Classification of Lesion

For evaluating the performance of skin lesion classification, evaluation metrics such as Sensitivity (*Sn*), Specificity (*Sp*), Accuracy (Ac), Dice index coefficient (*Dc*), and Jaccard score (*Js*) are used. These standardized criteria of evaluation are utilized for the validation of segmentation and classification performance in the ISBI challenge, which is considered to be a standard platform for publishing practical implementation of diagnosis of a melanoma lesion. Sensitivity (*Sn*) represents the ratio of accurate detection/segmentation of dermoscopic lesion, whereas Specificity (*Sp*) indicates the proportion of accurate detection/segmentation of non-melanoma pixels. Overall performance of diagnosis is quantified by a measure of the Accuracy (*Ac*) metric. The dice index coefficient (*Dc*) is used to measure the performance of detection/segmentation by comparing ground truth results. Similarly, the Jaccard score (*Js*) is a measure of the intersection of the union of segmented lesion with ground truth results. The area under curve (*AUC*) metric is used for assessing the performance by calculating the area of the ROC curve. A high value of *AUC* represents better classification architecture for the prediction of true values as true and false entities as false. Precision is the accuracy of the classification method to generate only valuable data, that is, it determines the rate of accurate detection. On the other hand, recall is the ability of the classifier to detect true values as true, which is also named the true positive rate. The weighted mean of recall and precision is termed the *F1* score or harmonic mean. *F1* is used to measure accuracy of machine learning architecture in a single score metric by evaluating both recall and precision. The Matthew correlation coefficient (*MCC*) quantifies the correlation between segmented and annotated area of lesion, the outcome of MCC ranges from −1 to 1. A larger value of *MCC* represents efficient detection/segmentation of skin lesions. The mathematical representation of all these matrices is shown as follows:

(19) $$Sn=\frac{TP}{TP+FN}$$

(20) $$Sp=\frac{TN}{TN+FP}$$

(21) $$Dc=\frac{2*TP}{\left(2*TP\right)+FP+FN}$$

(22) $$Js=\frac{TP}{TP+FN+FP}$$

(23) $$Ac=\frac{TP+TN}{TP+FN+TN+FP}$$

(24) $$AUC=\frac{\left(Sn+Sp\right)}{2}$$

(25) $$Pression=\frac{TP}{TP+FP}$$

(26) $$Recall=\frac{TP}{TP+FN}$$

(27) $$F1=2\;*\;\frac{Precision\;*\;Recall}{Precision+Recall}$$

(28) $$MCC=\frac{TP\;*\;TN-FP\;*\;FN}{\sqrt{\left(TP+FP\right)\left(TP+FN\right)\left(TN+FP\right)\left(TN+FN\right)}}$$

## 3. Results

This section comprises a detailed performance analysis of the proposed method. A machine with a Core-i7 processor and 32 GB of RAM is used to implement the proposed method and conduct all the experiments. OpenCV framework is used for processing and classification of the acquired images with Python programming language. Different parameters are used for analysis of performance at various stages of classification, namely evaluation of skin refinement efficiency, performance analysis of lesion localization, segmentation analysis, and evaluation of classification model. Efficiency of the proposed method is evaluated over five publicly available dataset: PH2, ISIC 2017, ISIC 2018, and ISIC 2019.

Pre-processing of skin lesions is one of the most important and vital stages in lesion classification. Efficient refinement of skin lesion enhances the classification results, as the pre-processing phase not only modifies the contrast and brightness of dermoscopic image, but also digitally removes the artifacts, which might end up misleading the classification result. Table 2 illustrate the performance of removal of artifacts digitally using proposed method for the PH2, ISIC 2017, ISIC 2018, and ISIC 2019 datasets, respectively. Evaluation metrics such as PSNR, MSE, RMSE, and UIQI are used to evaluate the efficiency of pre-processing. A low value of MSE and RMSE signifies robust image enhancement. The loss of energy in the pre-processed image is depicted by the value of RMSE. It evaluates the difference in intensity of the pre-processed and acquired image. A low value of RMSE portrays less distortion of the processed image. If the empirical score of PSNR is higher than 20 dB, it is a well-enhanced image. The quality of information sustained in the pre-processed image is portrayed by PSNR; higher values of PSNR depicts more sustained valuable details after removal of artifacts in the pre-processed image. The value of UIQI ranges from −1 to 1, which is used to ensure the image quality after enhancing the image. This matrix is used to express the evident change of removal of digital artifacts, such as hair, and thereafter refining the image from natural and clinical artifacts, such as clinical color swatches, clinical ruler marks, black frame, etc. Image quality is measured by UIQI with respect to human vision by employing parameters such as structural information, contrast, and luminance. Thus, it is used to measure the capacity of pre-processing method to remove the artifacts of images along with enhancing the image by modifying its contrast and sharpness, without lowering the image quality.

**Figure 7:**
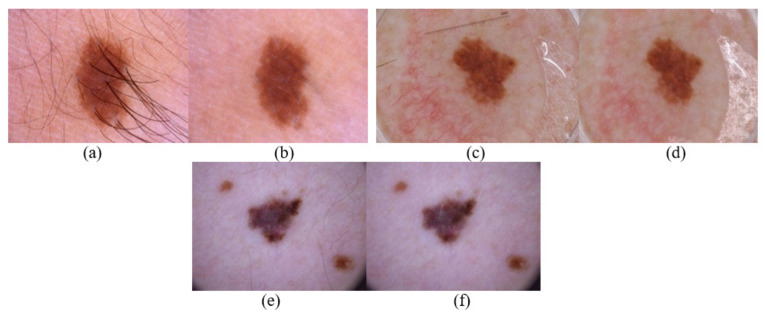
Illustration of proposed pre-processing for various artifacts. (**a**) ISIC_0000115 image with thick hair follicle. (**b**) Pre-processed output. (**c**) ISIC_0012395 image with ruler mark. (**d**) Pre-processed output. (**e**) ISIC_0024315 image with thin hair follicles. (**f**) Pre-processed output.

Table 2 show a highly acceptable range of PSNR (in Db) and UIQI, which represent the high-quality enhancement of the images in the pre-processing phase. Figure 7 pictorially represents the pre-processing phase, where artifacts such as thick and thin hair, black frame, ruler mark, and unwanted reflections are removed digitally by the proposed method of pre-processing. Table 3 show the classification of skin lesions with and without undergoing a pre-processing and segmentation phase; the accuracy mark illustrated in the tables for each dataset clearly concludes and supports the importance of a pre-processing and segmentation phase in computer-aided diagnostic systems. The above-mentioned tables show an enhanced accuracy score of about 8% to 9% when the acquired image is pre-processed. The F1 score, AUC, and MCC do represents the supremacy of performance when data are augmented properly before training and pre-processed and segmented before testing the model. Training and testing time for each of the datasets are tabulated in the Table 3. The training time for both the Table 3 (for all the datasets) are nearly same as size of the training data is the same (as ordinary data augmentation technique is implemented for data in Table 3. The above-mentioned experimental results and pictorial representation illustrates the need and importance of pre-processing images before classification.

Localization of suspected lesion is performed by the proposed classifier, which forms bounding box around the lesion and marks it to be ROI. Efficiency of localization is measured by metrics, such as mAP and IOU. The acceptable range of IOU for accurate area localization lies from 0.5 to 1. This metric is used to compare the predicted area with the ground truth, which is generated by expert dermatologists, to calculate the efficiency of localization. Table 4 represents the performance analysis of lesion localization by our proposed classifier on various dermoscopic datasets. The classifier successfully fetches an accuracy of 100% for PH2 and ISIC 2017 for skin lesion localization, which symbolizes the effectiveness of the modified layers of the classifier. Furthermore, the ISIC 2018 and ISIC 2019 datasets yield an accuracy of 99.40% and 99.56%, respectively. This enhanced tabulated result shows the accuracy of the classifier, and it provides a clear indication that the proposed classifier is capable to fetching improved results of classification of dermoscopic images.

The pre-processing phase is followed by the segmentation phase, whose efficiency is calculated and compared over parameters such as Sensitivity, Specificity, Dice score, Jaccard index, and Accuracy. An analysis of performance, based on these parameters, was proposed by ISIC, which is the official organization for publishing free accessible dermoscopic images for research and analysis on skin cancer. It is also mentioned by [23] that segmentation is a vital stage in CAD, which helps to improve the classification score. Table 5 encapsulates the analysis of segmentation performance over different datasets by the proposed method using neutrosophic and determinant methods. The novel proposed system seems to fetch an accuracy mark of 99.00% for the PH2 dataset, 98.83% for the ISIC 2017 dataset, 98.56% for the ISIC 2018 dataset, and 97.86% for the ISIC dataset. The system successfully fetches the sensitivity score (rate of accurate segmentation of true positives) of 97.56% for ISIC 2019, which contains visually challenging melanoma images. Similarly, the efficiency of the proposed method is reflected when it attains a mark of 97.97% of specificity (rate of accurate segmentation of false negatives) for ISIC 2019, which has a maximum number of different skin lesion classes, which makes it the most challenging dataset for even an expert dermatologist to perform accurate segmentation.

Efficient mathematical logic of uncertainty principle by neutroscopy assists the proposed system to fetch accurate and out-topping score for segmentation of lesion over state-of-the-art methods. Table 6 illustrates a comparison between the proposed method (PM) for segmentation and well-established state-of-the-art techniques. The segmentation performance of the proposed work is compared with the most inspiring methods for segmentation of dermoscopic images from the PH2 dataset. Reference [24] used a two-stage segmentation model by employing L-R fuzzy logic and graph theory to yield efficient segmentation results, while reference [25] employed a framework which works on the principal of semantic segmentation model for automatic segmentation. Moreover, reference [26] used alternate segmentation and classification by bootstrapping the DCNN model. The grab cut algorithm, which is semi-automatic in nature, was used by reference [27], whereas a deep convolutional neural network was employed for segmentation by reference [1]. We have also drawn inspiration for efficient segmentation models, which are proposed by references [28,29], where FCN networks and multistage fully convolution network (FCN) with parallel integration (mFCN-PI) is used for segmentation of dermoscopic lesion. However, our proposed method yields 99%, 97.50%, 99.36%, 95.12%, and 97.50% for accuracy, sensitivity, specificity, Jaccard score, and dice index, respectively, which are highest scores when compared with state-of-the-art methods.

A comparison of the proposed method against recently published and well-known segmentation methods for the ISIC 2017 dataset is tabulated in Table 7. The proposed method is contrasted against state-of-the-art methods for segmentation, such as the one used in reference [30], which utilizes an extension and amendment of FCN architecture, i.e., a fully convolutional residual network (FCRN). A robust deep learning SLS model of the encoder-decoder network was used in reference [31], where the dilated residual layers form the encoder network and decoder layer was constructed by a pyramidal pooling network, which was followed by three layers of convolution. Ref. [32] proposed a simultaneous segmentation and classification model using FrCN to yield a high score of specificity for the ISIC 2017 dataset. When the proposed segmentation method is computed for the ISIC 2017 dataset, it yields an accuracy mark of 98.83%, while other well-known and published works managed to obtain 97.33%, 95.30%, 81.57%, 95.06%, 93.39%, and 93.60% mark, respectively [24,25,27,29,30,31].

Segmentation performance of our method is contrasted against recently published segmentation methods, where Ref. [33] used a Difficulty-Guided Curriculum Learning (DGCL), Ref. [34] employed a Deep Saliency Segmentation method which employs a custom CNN of 10 convolutions, and Ref. [35] used AlexNet along with transfer learning for segmentation. Additionally, a few of the most successful segmentation models are proposed by [36], where the performance of U-Net is enhanced by BCDU-Net with convLSTM. Ref. [37] designed an architecture based on network of encoder and decoder for segmentation of skin lesion by Deep-Lab and PSP-Net; additionally, extraction of key features is performed by ResNet101. Despite using complex convolutions and architecture by these state-of-the-art methods, they cannot outperform the results of segmentation that is achieved by our method. Notwithstanding the fact that the proposed system yields an accuracy mark of 98.56%, our system not only fetches higher score for sensitivity (98.50%), but also shows its efficiency by yielding the highest specificity score of 98.58%. Table 8 represents a detailed comparison of the proposed segmentation method against state-of-the-art technology for the ISIC 2018 dataset.

Segmentation performance for the ISIC 2019 dataset is evaluated and compared against [23,38], which fetched accuracy scores of 96.74% and 93.98%, respectively, while our method yields 97.86%. The method proposed in [38] uses a triangular neutrosophic number and straight-line based method for segmentation. Dynamic thresholding using pentagonal neutrosophic method has been proven to work efficiently for all the datasets. Thus, an enhanced and modified pre-processing method combined with an advance segmentation method will yield a better performance for the classification of lesion, which is the fundamental aim of this research work. A tabular representation of the performance evaluation metrics is illustrated in Table 9.

Classification performance is evaluated for the proposed method (PM) and compared with other well-known classifiers, such as the YOLO, K-nearest neighbor (KNN), Support vector machine (SVM), Decision Tree (DT), Multilayer perceptron (MLP), Bayesian network (BN), random forest (RF), logistic, and naïve bayes (NB) algorithms. The performance is evaluated for Accuracy, Sensitivity, Specificity, Precision, and F1-Score to determine the efficiency of classification by different classifiers. The performance analysis is tabulated in Table 10 for PH2, ISIC 2017, ISIC 2018, and ISIC 2019. The proposed classifier proves to perform extremely well for all four datasets and the statistical figure proves the proposed classifier to be much more accurate than any other classifiers that are present in the state-of-the-art methods. The proposed classifier seems to score 99.50%, 99.33%, 98.56%, and 98.04% for the classification of the dermoscopic lesion from PH2, ISIC 2017, ISIC 2018, and ISIC 2019. Although the sensitivity score of the YOLO classifier for the ISIC 2019 dataset is the best out of all classifiers (with those which are compared), it scores 97.11% as its sensitivity score, whereas the proposed classifier scores 96.67%. With such high-scoring accuracy marks, the modified classifier proves itself to be eligible for execution of the application in a real-life scenario by dermatologists for the diagnosis of skin lesions. The tabulated data clearly indicate the need for selecting inception and residual blocks with a softmax layer in the architecture of the proposed classifier for the classification of malignant melanoma. 

## 4. Discussion

**Figure 8:**
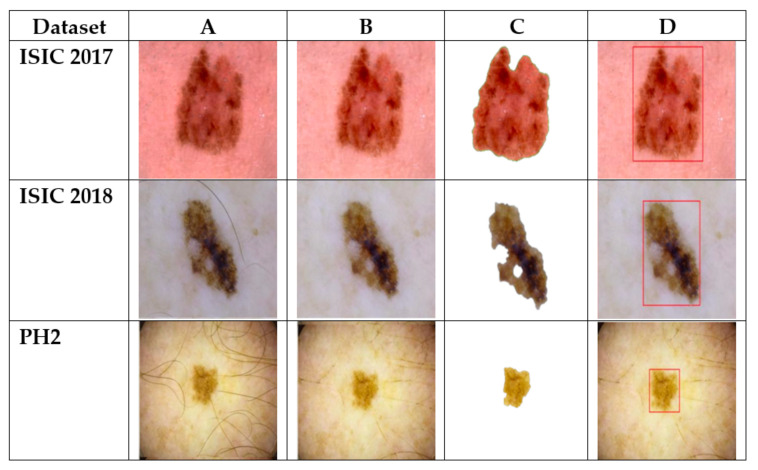
Detailed workflow of proposed system. (**A**) Image acquisition. (**B**) Pre-processing. (**C**) Segmentation. (**D**) Classification. The bounding box shows the detected lesion region.

The supremacy of the proposed classifier is evidently illustrated by the comparisons with well-known classifiers for the accurate identification of skin lesion. Our classifier yields a high score for all the evaluation parameters when compared with recently developed and extensively used classifiers. Due to its ability to distinguish the minor difference of pixel coloration for classification, it stands to be the most efficient and trustworthy for the classification of dermoscopic images. This article also focuses on the importance of an adequate pre-processing method for the removal of artifacts from the acquired image and thereafter enhancing the image, which will help the classifier to fetch such high values of accuracy. Notwithstanding the fact that segmentation of skin lesion using neutrosophy and a third-order determinant also helped to achieve high value of classification. The complete workflow of the proposed method is represented in Figure 8, where step ‘A’ shows the original dermoscopic image of the respective datasets, Step ‘B’ represents the pre-processed image, where digital artifacts are removed, and images are enhanced by the process of histogram equalization, Step ‘C’ portrays the segmentation phase where the lesion is segmented out from the dermoscopic image, and Step ’D’ is the final phase where the lesion is classified into either melanoma or non-melanoma and a bounding box is drawn around the lesion. Dermoscopic images from each dataset (the ISIC 2017, ISIC 2018, and PH2 datasets) are chosen to represent the workflow. Figure 8 shows the working accuracy of the proposed method, where each phase of the CAD system (preprocessing, segmentation, and classification) is accurately performed for a visually challenging dermoscopic image of melanoma lesion. 

**Figure 9:**
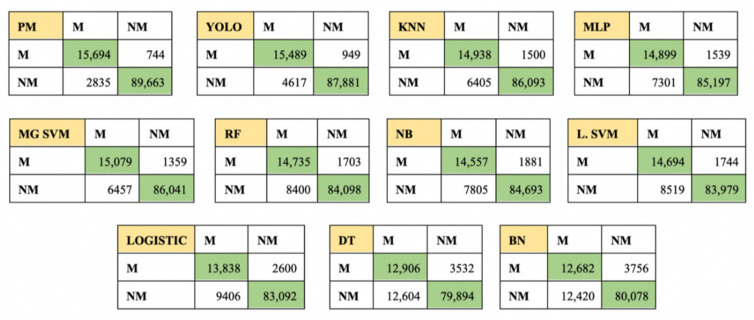
Confusion matrix for each classifier according to five-fold cross-validation.

The performance analysis of the classification of skin lesions using five-fold cross-validation is represented in Table 11, where performance metrics such as Accuracy (Acc), Sensitivity (Sen), Specificity (Spec), Precision (Prec), Recall (Rec), F1, MCC, AUC, and Standard deviation (SD) are calculated for different methods. A sum of 108,936 dermoscopic images are used to train and validate each method, out of which 103,524 images are from the training set (after data augmentation) and 5412 are from the validation set. The cross-validation fold is set to be 5, thereby avoiding any overfitting of data. In total, 21,787 dermoscopic images are randomly assigned to each of the four sample datasets. The performance of each model is based on the training on the first four sample datasets and validating it against the last sample dataset (of 21,787 images). The average performance of all these models is also encapsulated in Table 11 to show its overall performance. Table 11 concludes that the proposed method outperforms when trained and after five-fold cross-validation against the state-of-the-art models, such as KNN, YOLO, SVM, etc. A confusion matrix for each of the classifier is pictorially represented in Figure 9, which shows the value of the true positive, true negative, false positive, and false negative.

Statistical tests were conducted of a few of the dermoscopic skin lesion from the PH2, ISIC 2017, ISIC 2018, and ISIC 2019 datasets to check if the lesion can be classified into a specific class, based on statistical parameters, such as mean, skewness, and kurtosis. The mean value of an image represents the average pixel density, which can be an important parameter to classify a skin lesion as melanoma, because melanoma lesion does lie into specific colors ranging from bluish grey to dark brown. A mathematical representation of the mean value is shown in Equation (29), where *M* and *N* are dimensions of the image (I). The asymmetry distribution of pixels of a malignant lesion is market by *Skewness* of the image, which is mathematically represented in Equation (30), where μ is the mean of the distribution and σ is the standard deviation. The measure of tailedness of a pixel distribution is marked by *Kurtosis* (represented in Equation (31)) of an image. It is an important statistical parameter which shows that how often outliers occur.

(29) $$Mean=\frac{\sum_{x=1}^{M}\sum_{y=1}^{N}I\left(x,y\right)}{M\times N}$$

(30) $$Skewness=\frac{\sum_{x=1}^{M}\sum_{y=1}^{N}{\left(I\left(x,y\right)-\mu \right)}^{3}}{M\times N\times \sigma^{2}\;}$$

(31) $$Kurtosis=\frac{\sum_{x=1}^{M}\sum_{y=1}^{N}{\left(I\left(x,y\right)-\mu \right)}^{4}}{M\times N\times \sigma^{4}\;}$$

A statistical comparison of eight different dermoscopic images from the PH2, ISIC 2017, ISIC 2018, and ISIC 2019 datasets based on statistical parameters such as mean, skewness, and kurtosis is tabulated in Table 12. The tabular values show a higher value of the mean for images which belong to the melanoma class, which is because a melanoma lesion visually appears to be more darkish than a non-melanoma lesion. Similarly, a trend towards a higher value for melanoma lesion images can be noticed for skewness, with a melanoma being asymmetry in shape showing a more random distribution of pixels (i.e., higher value for skewness) than its counterpart. It is also noticed that the kurtosis value is very pointy (high value) for non-melanoma lesions and a bit flat (lower value) for the melanoma image. Notwithstanding the fact that there are few exceptions, such as for ISIC_0034412, which is a non-melanoma image, the mean value is as high as 201.184, which is because of the visually dark appearance of non-melanoma lesions. However, due to visual similarities of both the lesions, it is statistically a bit difficult to classify this lesion, but with the help of deep neural networks, we can classify them accurately, as the features are mapped for each lesion.

The mean accuracy obtained using the proposed method was higher than that for the state-of-the-art techniques. However, to assess how significantly different the accuracy is, we also performed a statistical analysis. The statistical test for all the classifiers based on the test datasets is encapsulated in Table 13. The test dataset contains 4450 dermoscopic images, which are taken from the ISIC 2017, ISIC 2018, and ISIC 2019 repositories. The *p*-value is calculated using the Freidman test with Bergmann and Hommel’s correction. If the value of ‘p’ is less than 0.05, then it shows that the null hypothesis (H_0_) is proven wrong, thereby proving the alternative hypothesis (H_a_), which states that the proposed algorithm outperforms all of the state-of-the-art classifiers, as there exists a significant difference in the mean accuracy. H_0_ is used to prove that there exists no difference in the mean accuracy of the classifiers when the models are classified using the test dataset. The *t*-test is performed because the classifiers run over the same dataset, thereby producing a *t*-value. The performance of each classifier is compared in a pair-wise manner against the proposed method. Hence, the first row of the table contains not-a-number (NaN). The mean rank for each classifier is calculated by ranking the performance of classification for each image in the test dataset by several classifiers (line KNN, MGSVM, etc.). The proposed method outperforms in classification all the dermoscopic test images; thereby, its mean rank (sum of ranks for all the images in test dataset/total image in test dataset) is 1.12. The mean and standard deviation of confidence value (the probability of a true or false value during classification) for each dermoscopic image in the test dataset is encapsulated under the mean and SD columns in Table 13. A low SD value shows an equal confidence score for all the images in the test dataset, which shows that the hyperparameters are tuned properly, as there exists a minor variance in the classification score. The results in Table 13 clearly depict the statistical supremacy of the proposed method over well-known classifiers. The *p*-value for all the classifiers is very low, which signifies the mean accuracy difference; moreover, the mean confidence score of the proposed method is 0.86, which is higher than any existing method, thereby portraying the efficiency of the proposed method.

## 5. Conclusions

In this article, an effective pre-processing model was proposed to digitally remove the artifacts present in the acquired image; the image is enhanced by the method of histogram equalization. After refining the image, it undergoes the segmentation phase, where a mathematical-based algorithm using thresholding and pentagonal neutrosophy is demonstrated to achieve enhanced segmentation results. Henceforth, the segmented image is classified using the proposed classifier. The model was trained with a huge dataset, which was proposed after data augmentation and balancing. The outcomes of the proposed methods were evaluated using publicly accessible datasets: PH2, ISIC 2017, ISIC 2018, and ISIC 2019. A vivid and vast range of test parameters proved that the proposed methods, for each stage of CAD, outperformed the state-of-the-art methods. The proposed method had a significantly higher score in sensitivity and specificity in the field of diagnosis of melanoma lesion. The classification results mentioned in Table 10 were end-to-end deep learning models, i.e., each dataset was augmented (by the proposed augmentation technique), and the training images underwent the process of pre-processing and segmentation before the classification of the skin lesion. Various state-of-the-art classifiers, such as YOLO, KNN, Bayesian networks, etc., were employed on the same augmented and processed data. Our proposed deep neural network outperformed all the state-of-the-art classifiers for classification of skin lesion images. The importance of the proposed data-augmentation technique, pre-processing, and segmentation is highlighted in Table 3 where the proposed deep neural network is used to classify the skin lesion (with the standard data augmentation technique only); however, it underperformed when compared to the model which employed the proposed techniques for data augmentation, pre-processing, and segmentation. The sensitivity and specificity scores of the model which employed the proposed techniques seem to be 8–10% higher than the model with standard augmentation.

More pronounced output can be achieved in the near future, when an extensive and varied range of datasets with several classes will be available, along with modified CAD equipment, which will increase the image quality during image acquisition. A broad range of classifications for various skin diseases (such as Vitiligo, Alopecia areata, Psoriasis, Atopic dermatitis, and Lamellar ichthyosis) and several other types of skin cancers (such as basal cell carcinoma, squamous cell carcinoma, actinic keratoses, etc.) should be performed in future research. In future research, we also aim to deploy the proposed algorithms into a smartphone application, such that the diagnosis of skin cancer is made easily available without the need for any invasive techniques. Additionally, we also aim to proposes a smartphone-based dermoscopic tool which will increase the accuracy of diagnosis when diagnosed using a smartphone. The digital diagnosis of skin lesions is an extensive area of research and it has a great potential in the near future.

## Figures and Tables

**Table 1:** Dataset distribution of different datasets that are employed in this research.

Dataset	Training			Training Samples	Testing			Validation			Total	
	M	NM	T	(Train ×4)	M	NM	T	M	NM	T	M	NM
PH2	0	0	0	0	0	0	0	40	160	200	40	160
ISBI17	374	1626	2000	8000	197	403	600	30	120	150	1723	7057
ISIC18	779	7916	8695	34,780	334	986	1320	0	0	0	3450	32,650
ISIC19	2713	12,473	15,186	60,744	905	4158	5053	904	4158	5062	12,661	58,208

**Table 2:** Evaluation of the pre-processing performance of different datasets.

Dataset	Artifacts	PSNR (dB)	MSE	RMSE	UIQI
PH2	Thick hair	39.37	34.69	5.89	0.64
	Thin hair	40.53	12.57	3.57	0.67
	Ruler Marks	37.76	83.54	9.14	0.68
	Black frame	39.64	58.98	7.68	0.65
	Color patch	38.99	46.51	6.82	0.70
ISIC 2017	Thick hair	35.70	37.58	6.13	0.59
	Thin hair	37.58	32.38	5.69	0.62
	Ruler Marks	32.67	115.99	10.77	0.60
	Black frame	36.00	98.61	9.93	0.61
	Color patch	35.79	55.80	7.47	0.63
ISIC 2018	Thick hair	31.75	62.41	7.90	0.55
	Thin hair	34.56	62.25	7.89	0.59
	Ruler Marks	32.56	113.42	10.65	0.57
	Black frame	34.67	129.28	11.37	0.60
	Color patch	33.13	92.54	9.62	0.58
ISIC 2019	Thick hair	30.78	110.67	10.52	0.50
	Thin hair	33.24	71.91	8.48	0.55
	Ruler Marks	29.45	180.37	13.43	0.55
	Black frame	33.45	218.45	14.78	0.50
	Color patch	32.09	104.45	10.22	0.57

**Table 3:** Classification of results with and without undergoing pre-processing and segmentation stage. Accuracy, sensitivity, and specificity are represented as Ac, Sn, and Sp, respectively. Training and testing time are represented as Tr. Time (in hours) and T. Time (in sec), respectively.

Datasets	Ac (%)	Sn (%)	Sp (%)	F1 (%)	AUC (%)	MCC (%)	Tr. Time	T. Time
**Without pre-processing**								
PH2	91.00	87.50	91.88	79.55	89.69	74.34	0.0	10.09
ISIC 2017	91.50	89.34	92.56	87.35	90.95	81.01	5.25	18.41
ISIC 2018	90.53	89.22	90.97	82.62	90.10	76.57	21.67	24.80
ISIC 2019	90.35	88.67	90.94	82.69	89.80	76.38	46.33	129.99
**With pre-processing**								
PH2	99.50	100	99.38	98.77	99.68	98.46	0.00	9.35
ISIC 2017	99.33	98.48	99.75	98.98	99.12	98.49	5.33	17.22
ISIC 2018	98.56	97.61	98.88	97.17	98.25	96.21	21.75	26.97
ISIC 2019	98.04	96.67	98.52	96.24	97.59	94.91	46.17	110.83

**Table 4:** Performance of lesion localization is evaluated for the PH2, ISIC 2017, ISIC 2018, and ISIC 2019 datasets.

Datasets	Sensitivity (%)	Specificity (%)	mAP	IOU
PH2	100	100	0.94	100
ISIC 2017	100	99.75	0.98	98.99
ISIC 2018	99.40	99.90	0.98	98.21
ISIC 2019	99.56	99.77	0.97	97.79

**Table 5:** Performance analysis of the segmentation of the PH2, ISIC 2017, ISIC 2018, and ISIC 2019 datasets.

Datasets	Accuracy (%)	Sensitivity (%)	Specificity (%)	Jac (%)	Dic (%)
PH2	99.00	98.48	99.38	96.52	98.23
ISIC 2017	98.83	98.50	99.01	94.54	97.19
ISIC 2018	98.56	97.56	98.58	92.23	97.50
ISIC 2019	97.86	97.50	97.97	95.12	95.97

**Table 6:** Comparison of the proposed segmentation method with the state-of-the-art methods for the PH2 dataset.

Reference	Accuracy (%)	Sensitivity (%)	Specificity (%)	Jac (%)	Dic (%)
PM	99.00	97.50	99.38	95.12	97.50
[24]	97.50	97.50	95.50	88.64	93.97
[25]	98.70	92.90	96.90	----	----
[26]	96.50	96.70	94.60	89.40	94.20
[27]	92.99	83.63	94.02	79.54	88.13
[1]	95.41	----	----	----	----
[29]	95.03	96.23	94.52	85.90	92.10
[28]	94.24	94.89	93.98	83.99	90.66

**Table 7:** Comparison of the proposed segmentation method with state-of-the-art methods for the ISIC 2017 dataset.

Reference	Accuracy (%)	Sensitivity (%)	Specificity (%)	Jac (%)	Dic (%)
PM	98.83	98.48	99.01	96.52	98.23
[24]	97.33	91.45	98.76	86.99	93.04
[25]	95.30	87.5	85.5	----	----
[30]	81.57	75.67	80.62	----	----
[29]	95.06	86.05	95.95	79.15	88.95
[27]	93.39	90.82	92.68	74.81	84.26
[31]	93.60	81.6	98.3	78.2	87.8
[32]	93.20	82.00	97.80	76.20	84.70

**Table 8:** Comparison of the proposed segmentation method with state-of-the-art methods for the ISIC 2018 dataset.

Reference	Accuracy (%)	Sensitivity (%)	Specificity (%)	Jac (%)	Dic (%)
PM	98.56	98.50	98.58	94.54	97.19
[33]	94.80	89.10	96.40	80.70	88.10
[34]	92.69	----	----	----	----
[30]	81.79	81.8	71.4	----	----
[35]	98.70	95.60	99.27	----	----
[36]	93.70	78.50	98.20	93.70	----
[37]	94.20	90.60	96.30	83.80	89.80

**Table 9:** Comparison of the proposed segmentation method with state-of-the-art methods for the ISIC 2019 dataset.

Reference	Accuracy (%)	Sensitivity (%)	Specificity (%)	Jac (%)	Dic (%)
PM	97.86	97.56	97.97	92.23	95.96
[38]	96.74	94.69	97.34	86.81	92.94
[24]	93.98	91.55	94.84	79.84	88.79

**Table 10:** Evaluation of the classification performance (in %) of different classifiers.

**PH2 Dataset**							
**Reference**	**Accuracy**	**Sensitivity**	**Specificity**	**Precision**	**F1**	**AUC**	**MCC**
PM	99.50	100	99.38	97.56	98.77	99.69	98.46
YOLO	97.50	97.50	97.50	90.70	93.98	97.50	92.50
KNN	94.00	95.00	93.75	79.17	86.36	94.38	83.12
MLP	95.00	95.00	95.00	82.61	88.37	95.00	85.55
MG SVM	93.50	90.00	94.38	80.00	84.71	92.19	80.82
RF	90.00	82.50	91.88	71.74	76.74	87.19	70.69
NB	90.00	92.50	89.38	68.52	78.72	90.94	73.77
LINEAR SVM	92.50	90.00	93.13	76.60	82.76	91.56	78.42
LOGISTIC	90.50	87.50	91.25	71.43	78.65	89.38	73.24
DT	87.00	82.50	88.13	63.46	71.74	85.31	64.40
BN	89.50	85.00	90.63	69.39	76.41	87.81	70.33
**ISIC 2017 dataset**							
**Reference**	**Accuracy**	**Sensitivity**	**Specificity**	**Precision**	**F1**	**AUC**	**MCC**
PM	99.33	98.48	99.75	99.49	98.98	99.12	98.48
YOLO	98.33	96.95	99.01	97.95	97.45	97.98	96.21
KNN	97.00	94.92	98.02	95.90	95.41	96.47	93.18
MLP	95.67	92.39	97.27	94.30	93.33	94.83	90.13
MG SVM	93.67	89.85	95.53	90.77	90.31	92.69	85.61
RF	93.67	88.33	96.28	92.06	90.16	92.30	85.53
NB	90.67	86.29	92.80	85.43	85.86	89.55	78.90
LINEAR SVM	88.17	85.28	89.58	80.00	82.56	87.43	73.70
LOGISTIC	90.83	86.29	93.05	85.86	86.08	89.67	79.24
DT	88.50	81.22	92.06	83.33	82.26	86.64	73.77
BN	85.17	80.20	87.59	75.96	78.03	83.90	66.89
**ISIC 2018 dataset**							
**Reference**	**Accuracy**	**Sensitivity**	**Specificity**	**Precision**	**F1**	**AUC**	**MCC**
PM	98.56	97.61	98.88	96.74	97.17	98.24	96.21
YOLO	97.50	96.11	97.97	94.14	95.11	97.40	93.44
KNN	96.36	93.11	97.47	92.56	92.84	95.29	90.40
MLP	95.08	89.82	96.86	90.63	90.23	93.34	86.94
MG SVM	93.64	86.53	96.05	88.11	87.31	91.29	83.07
RF	93.64	87.72	95.64	87.20	87.46	91.68	83.20
NB	90.91	87.13	92.19	79.08	82.91	89.66	76.90
LINEAR SVM	86.52	83.54	87.52	69.40	75.82	85.53	67.13
LOGISTIC	88.79	84.73	90.16	74.47	79.27	87.45	71.91
DT	87.50	81.14	89.66	72.65	76.66	85.40	68.36
BN	83.71	83.23	83.87	63.62	72.11	83.55	61.70
**ISIC 2019 dataset**							
**Reference**	**Accuracy**	**Sensitivity**	**Specificity**	**Precision**	**F1**	**AUC**	**MCC**
PM	98.04	96.67	98.52	95.82	96.24	97.59	94.91
YOLO	97.92	**97.11**	98.20	95.00	96.04	97.66	94.63
KNN	97.11	95.33	97.73	93.67	94.49	96.53	92.54
MLP	95.49	93.56	96.17	89.57	91.52	94.86	88.49
MG SVM	94.10	91.33	95.08	86.71	88.96	93.21	85.01
RF	94.05	94.67	93.83	84.36	89.21	94.25	85.39
NB	87.40	89.11	86.80	70.35	78.63	87.95	70.85
LINEAR SVM	90.58	93.56	89.53	75.86	83.78	91.54	78.09
LOGISTIC	90.52	88.44	91.25	78.04	82.92	89.85	76.68
DT	89.31	90.89	88.75	73.96	81.55	89.82	74.92
BN	84.80	87.33	83.91	65.61	74.93	85.62	65.69

PM = Proposed method.

**Table 11:** Evaluation of the classification performance for different models according to five-fold cross-validation.

Classifier	Acc	Sen	Spec	Prec	Rec	F1	MCC	AUC	SD
PM	96.72	95.47	96.93	84.70	95.47	89.76	88.03	96.20	9.31
YOLO	94.89	94.23	95.01	77.04	94.23	84.77	82.33	94.62	7.71
KNN	92.74	90.88	93.07	69.99	90.87	79.08	75.71	91.98	3.41
MLP	91.89	90.64	92.11	67.11	90.64	77.12	73.53	91.37	3.12
MG SVM	92.83	91.73	93.02	70.02	91.73	79.42	76.17	92.38	4.51
RF	90.73	89.64	90.92	63.69	89.64	74.47	70.51	90.28	1.83
NB	91.11	88.56	91.56	65.09	88.56	75.04	71.01	90.06	0.44
LINEAR SVM	90.58	89.39	90.79	63.31	89.39	74.12	70.08	90.09	1.51
LOGISTIC	88.98	84.18	89.83	59.53	84.18	69.74	64.66	87.01	5.17
DT	85.19	78.51	86.37	50.59	78.51	61.53	54.84	82.44	12.44
BN	85.15	77.15	86.57	50.52	77.15	61.05	54.16	81.86	14.18

PM = Proposed method; SD = Standard deviation.

**Table 12:** Evaluation of statistical tests for dermoscopic images.

Image	Mean	Skewness	Kurtosis	Prediction	Class
ISIC_0012425	208.79	0.96	−1.09	1	Melanoma
ISIC_0012448	140.30	−0.93	2.93	0	Non-Mel
ISIC_0012551	93.87	−0.17	4.66	0	Non-Mel
ISIC_0013072	154.29	−0.17	1.90	1	Melanoma
ISIC_0034329	217.14	0.52	2.34	1	Melanoma
ISIC_0034412	201.18	−0.79	10.14	0	Non-Mel
ISIC_0034343	197.83	−1.42	4.90	1	Melanoma
ISIC_0034520	166.67	−0.69	8.17	0	Non-Mel

**Table 13:** Pair-wise statistical test of the proposed method (PM) compared to other classifiers.

Classifier	*t*-Value	*p*-Value	Mean Rank	Mean	SD
PM-PM	NaN	NaN	1.12	0.86	0.0670
PM-YOLO	1.61	0.0183	2.00	0.83	0.0830
PM-KNN	5.75	0.0045	2.92	0.77	0.0717
PM-MLP	16.90	0.0719	7.19	0.69	0.0723
PM-MG SVM	6.10	0.0037	3.82	0.75	0.0730
PM-RF	16.22	0.0085	7.86	0.66	0.0714
PM-NB	11.49	0.0003	4.94	0.73	0.0621
PM-LSVM	11.06	0.0004	6.06	0.70	0.0620
PM-LOGISTIC	16.69	0.0075	9.16	0.63	0.0629
PM-DT	15.86	0.0092	10.11	0.63	0.0685
PM-BN	17.96	0.0056	10.78	0.60	0.0647
